# Remarkable influence of microwave heating on Morita-baylis-Hillman reaction in PEG-200

**DOI:** 10.1186/1752-153X-6-30

**Published:** 2012-04-11

**Authors:** A Aravind, Sanil George, Santhosh Kumar

**Affiliations:** 1 Chemical Biology Laboratory, Division of Molecular Medicine and Disease Biology, Rajiv Gandhi Centre for Biotechnology, Thiruvananthapuram, Kerala, 695014, India

## Abstract

**Background:**

Morita Baylis Hillman (MBH) reaction is used to introduce carbon-carbon or carbon-heteroatom bond in a molecule. The major drawback of this reaction is the relatively low product yield and long reaction time. Though notable changes have been made to improve the reaction rate and yield of MBH adduct by various groups, a reliable synthetic procedure under ambient temperature in presence moisture and air is remain unsolved. Continuing the effort to improve the rate and yield, we report here an eco-friendly and cost-effective method to generate MBH adducts. Non-volatile polyethylene glycol-200 is used as reusable solvents and the reaction was carried out under the influence of microwave energy.

**Results:**

Microwave irradiation have a remarkable influence on PEG suspended 4-Diazabicyclo [2.2.2] octane (DABCO) catalysed MBH reaction between aldehydes and ethyl acrylate. Molecular weight of the PEG is found to have a significant influence on the reaction yield. PEG-200 was the most efficient solvent and in combination with DABCO, the medium can be recycled upto three more runs. This reaction condition is successfully applied to obtain MBH adduct of five different aldehydes in very short time with excellent yield and the required catalyst concentration was very low compared to standard MBH reaction. Since the MBH adduct is an important reactive intermediates for many complex organic syntheses, this approach can be successfully utilised as an alternative to existing reaction conditions.

**Conclusion:**

A new method was developed to improve the reaction rate and yield of MBH reaction The PEG 200-DABCO combination provides a sustainable, non-volatile, recyclable and environment friendly solvent medium to produce MBH adducts. This medium in combination with microwave energy proved to be very effective to introduce a new carbon-carbon or a carbon-heteroatom bond in a molecule.

## Background

Morita Baylis-Hillman reaction is one of the most popular synthetic techniques used by the organic chemists to introduce a carbon-carbon or a carbon-heteroatom bond in a molecule. The synthetic adduct formed can be used as an important intermediate for many multi-organic syntheses [1-3]. The reaction involves the coupling of α, β unsaturated carbonyl compound with an aldehyde in the presence of a nucleophilic catalyst such as tertiary amines or phosphine. Though this technique has been successfully utilized in many organic syntheses, several drawbacks that includes relatively low product yield, extended time of reaction, high concentration of the mild base catalyst and requirements of hazardous organic solvents have hampered its effective utilisation. Because of the synthetic potential of this reaction, several modifications have been suggested by various research groups around the world to enhance the reaction rate and yield of the synthetic adduct [4-8]. The reported modified procedures includes the use of several new catalysts [9-13], solvent media [14-17], performing the reaction under high pressure [18-20], exposure to ultrasound energy [21] etc. It is also reported that a combination of mild base catalyst and an ionic liquid solvent can enhance the rate of formation of the addition product. Though most of these refinements enhance the reaction rate and product yield, it is limited only to certain substrate molecules. At present, the widely accepted general and practical approach to increase the product yield is by using very high concentration of the reactants and stoichiometric excess of the catalyst [22-26]. This is not advisable from the economic and environmental perspective and it is necessary to recycle or recover the catalyst after each reaction. Though the catalyst incorporated polymer-supported procedure [27-29] has been tried recently to prevent the loss of catalyst, it met with only limited success. Recyclable ionic liquid dual solvent-catalysts [30-34] that serves as solvent and catalyst were also reported with satisfactory results. Several of these refinements uses considerable amount of hazardous and costly organic chemicals that include the extraction and purification procedures, which is environmentally not idyllic.

Release of large amount of volatile organic solvents from the industries and laboratories into the environment posed a serious threat to the very existence of life on earth. Therefore various groups across the world are now exploring the feasibility of various alternate reaction procedures to reduce the release of these hazardous chemicals into our environment. Microwave assisted Organic Synthesis (MAOS), a “Green Chemistry” based synthetic procedure using eco-friendly liquids as solvent/co-solvent is very effective in accelerating the rate of several organic reactions [35,36]. The quest for eco-friendly solvent to the conventional organic solvents has resulted many “neoteric solvents” such as ionic liquid, supercritical carbon dioxide (sc CO_2_) [37,38] and polyethylene glycol and their solutions. In this category, polyethylene glycol (PEG) is the most popular solvent because of its low cost, low toxicity, reduced flammability, high thermal stability, recyclability and biological compatibility [39-41]. Degradable nature of the PEG and its ability to form homogenous mixture with a broad range of solvents make it as the best alternative solvent system for organic chemists.

We studied the effect of microwave radiation on MBH reactions in synthetic grade polyethylene glycol as solvent medium. PEG-200, PEG-300, PEG-400, PEG-1000 were used to study the effect of the solvent molecular weight on the reaction rate and the results were compared with conventional MBH reaction. PEG-200-DABCO combination was found to be the best medium that can effectively influence the formation of a new carbon-carbon bond in a molecule. Exposure of the reaction medium to microwave energy had a profound influence on MBH reaction and it helped to bring down the overall reaction time significantly. The results are discussed in this paper in detail.

## Results and discussion

**Scheme 1:**
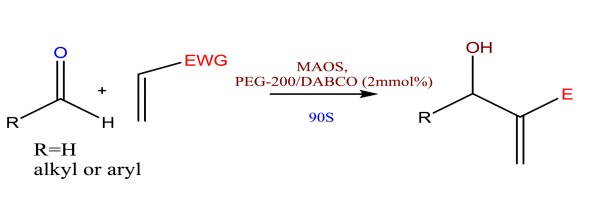
Synthesis of ethyl-2-(hydroxymethyl)acrylate.

Our effort was to synthesize a monomer molecule that serves as the basic building block to generate a multifunctional nanocarrier molecule as drug delivery vehicle by MBH reaction. This reaction was severely hampered by the very low intermediate product yield. We carried out DABCO catalysed addition reaction between ethyl acrylate and formaldehyde to produce ethyl-2-(hydroxymethyl) acrylate. Initially methanol and then THF were tried as the solvent medium (Scheme 1). However even after 48 h, the yield of the product was found to be very low (Additional file 1: Table  S1). Similar results were obtained with other polar solvents like dioxane and THF. This addition reaction was carried out using several aldehydes bearing electron donating and withdrawing groups to find out whether the choice of aldehyde and the solvent medium has any influence on product yield. The results showed that the substrate choice had only a limited influence on product yield. But when traditional organic solvents like THF, methanol, etc. were replaced with low molecular weight polyethylene glycol medium, the product yield showed a dramatic improvement and in all cases high product yield was achieved within first four hours of reaction.

Influence of the molecular weight of solvent PEG on MBH reaction was studied by performing the addition reaction in PEG-200, PEG-300, PEG-400, PEG-600 and PEG-1000. The results showed that though PEG was an effective solvent and its molecular weight was found to have a remarkable influence on the product yield. As the molecular weight decreases the product yield showed a significant increase and it was achieved in 4 h (Additional file 2: Table  S2, Additional file 3: Table  S3, Additional file 4: Table  S4, Additional file 5: Table  S5, Additional file 6: Table  S6). In low molecular mass the polyethylene oxides chain of PEG tends to converge into a small volume of space to form a compact conformation. This conformation may not be resulted from the hydrophobic interaction between the methylene groups present in the PEG. This conformation can effectively influence the interaction between the hydrophobic pockets of solvent and the solutes which may contribute a positive effect on the reaction rate. As the polyethylene oxide chain length increases, the molecule adopts a random coil conformation which may lead to a structured region around the non-polar part of the reactant. This can prevent the effective interaction between the reactants in the medium. In short, MBH reaction in low molecular weight PEG-DABCO medium helped to produce very high product yield, in most case within first four hours of reaction (Additional file 2: Table  S2).

The nature of the aldehydes that selected to carry out MBH reaction in PEG-200-DABCO medium had influenced the outcome of the reaction to some extent in terms of product yield. Reaction with benzaldehyde produced about 75% yield within the first 4 h of reaction (Additional file 3: Table  S3). In the case of electron rich aldehydes like 2-Methoxy benzaldehyde, the product yield was comparatively low under the same experimental conditions (Additional file 4: Table  S4). But with 4-chlorobenzaldehyde and 4-nitrobenzaldehyde, the addition product yield obtained was higher (Additional file 5: Table  S5, Additional file 6: Table  S6) compared to that of benzaldehyde and the time required to achieve the maximum yield was 4 h. A comparative analysis between the percentage conversion of the addition product and the reaction time clearly showed that molecular mass of PEG had a marked influence on overall product yield and PEG-200-DABCO medium is the best to carry out MBH reaction between the aldehydes and ethyl acrylate.

Recyclability of the PEG-200-DABCO medium was investigated by performing the above reactions in the next three subsequent runs to a fixed time period. After each run the product was recovered and the medium was used to perform the same reaction by adding fresh reactants and without adding the catalyst. The reaction was allowed to continue up to 4 h. But after the first run, ability of PEG-200-DABCO to act as an effective recyclable medium showed a steady decline in terms of product yield isolated during the subsequent runs. This loss in efficiency can be compensated to some extent by extending the overall reaction time (Additional file 7: Table  S7 and Additional file 8: Table  S8). After each run the product was recovered and the chemical integrity of the medium was analyzed using FT-IR and ^1^H NMR. The spectrum showed no significant additional spikes compared to that of original solvent used for the first run. In general the recycling medium DABCO/PEG-200 was confined only to same type of reaction and the effectiveness of the recycling concept was not yet substantiated with other type of MBH reactions carried out with different aldehydes considering the efforts required to recover the product after each run.

Exposure of the reaction medium to microwave radiation imbued a dramatic influence on overall yield of the reaction product and the required time to accomplish this is only 90 s. All the reactions were carried out at 60°C by keeping the pressure inside the reaction vessel at 175 psi. The reaction mixture was exposed to microwave radiation (300 W) and about 90% of the reactants were converted to addition product in ninety seconds. Similar results were obtained with all other aldehydes (Additional file 9: Table  S9) under the same experimental conditions. A comparative study of standard MBH reaction and microwave assisted addition reaction in PEG-200-DABCO medium showed that microwave energy has a profound effect on MBH reaction. Under the influence of micro wave energy high product yield was achieved in very short time and the required catalytic concentration was only about one tenth compared to that required to carry out standard MBH reaction.

Influence of the microwave energy on recycling medium after each recycling step was tested by performing the MBH addition reaction between the aldehydes and ethyl acrylate in PEG-200-DABCO medium. The exposed time for each case was 90s. After the first run the product was isolated and the medium was used for the next run by adding fresh reactants and without adding the catalyst. But after the first run the medium showed a steady decline in its efficiency in terms of product yield isolated during the next three runs. After each run, the colour of the medium slowly turned into a dark brown and its intensity increases with the subsequent run. But the FT-IR spectrum of the medium revealed no significant change in its molecular integrity. In any case, yield of the addition product obtained during the subsequent run under in the influence of microwave energy was higher compared to that in its absence (Additional file 10: Table  S10). All results showed that microwave energy had a profound influence in terms of product yield on the outcome of all MBH addition reactions between aldehydes and ethyl acrylate in PEG-200-DABCO medium.

## Experimental section

### General

All the microwave assisted organic reactions were performed in a CEM Discover microwave system in a 10 mL seamless pressure vials. The reaction temperatures were monitored by an equipped IR sensor. Glasswares were oven dried and cooled in a desiccator (P_2_O_5_ desiccant) prior to its use. All commercially available chemicals were used as-received. HPLC grade solvents were used for the synthesis. Reactions involving anhydrous conditions were conducted in dry glassware under nitrogen atmosphere. FT-IR spectra were recorded on a Nicolet^TM^ 5700 spectrophotometer (Thermo Electron Corporation) and ^1^H and ^13^ C NMR spectra were recorded on Bruker AMX 300 (^1^H: 300 MHz, ^13^ C: 100 MHz). Merck silica gel 60 (70–230 mesh) and (0.063–0.200 mm) were used for flash chromatography. Mass spectra were obtained on JOEL JMS 600 at an ionization potential of 70 eV. Analytical thin layer chromatography (TLC) was carried out using TLC silica gel 60 F254 plates. Plates were visualized by exposing to ultraviolet (254 nm) radiation.

### MBH reaction in methanol or THF using DABCO catalyst

Ethyl acrylate (1 mole), aldehydes (1.5 moles), DABCO (20 mmol%) were mixed in a flask, (pH 8.9). The mixture was stirred at 50°C. After 48 h, the pH of the solution was brought down to 5.5 by adding dilute HCl. The mixture was wriggled vigorously with diethyl ether (200 mL) and the organic phase was separated, washed with brine (50 mL) and dried over anhydrous sodium sulphate. Organic solvent was distilled off and the residue was fractionally distilled. The reactions were closely monitored by TLC. The resulting residue was purified by flash column chromatography and then by HPLC.

#### 3-ethoxy-2-methylenebut-3-en-1-ol

Colourless oil; IR (neat): *υ*_max_3408, 2878, 1056, 936, 885 cm^−1^: ^1^H NMR (300 MHz, CDCl_3_, 25°C), δ (ppm): 1.29 (t, 3H, CH_3_), 4.20 (q, 2H, CH_2_CH_3_), 5.85–6.15 (2 m, 2H, =CH_2_), 3.32 (s, 2H, CH_2_). ^13^ C NMR (CDCl_3_, 100 MHz): δ (ppm): 166.5 (C), 14.2 (C), 124.8 (CH_2_), 61.9 (CH_2_), 14.5 (CH_3_). MS: m/z 115.1 (M^+^-1).

### MBH reaction using PEG as solvent medium

Ethyl acrylate (3 mmol), aldehyde (2 mmol) and DABCO (20 mmol%) in was dissolved in respective PEG having different molecular weight in a RB flask. The reaction mixture was stirred continuously. After 4 h, product was extracted with dry ether (5X15 mL). The solvent ether was evaporated and the product was monitored by TLC. It was further purified by flash column chromatography.

#### 3-ethoxy-2-methylenebut-3-en-1-ol

Colourless oil; IR (neat): *υ*_max_3415, 2898, 1713, 1350, 1273, 1027, 943, 885 cm^−1^: ^1^H NMR (300 MHz, CDCl_3_, 25°C), ^1^H NMR (300 MHz, CDCl_3_, 25^0^ C), δ (ppm): 1.29 (t, 3H, CH_3_), 4.24 (q, 2H, CH_2_CH_3_), 5.58, 6.51 (2 m, 2H, =CH_2_), 4.23 (s, 2H, CH_2_OH), 5.51 (s, 1H, OH). ^13^ C NMR (100 MHz, CDCl_3_, 25°C), δ (ppm): 166.9 (C), 140.1 (C), 124.5 (CH_2_), 61.7 (CH_2_), 61.5 (CH_2_), 14.2 (CH_3_). MS: m/z 131.1 (M^+^+1).

### Procedure for the recyclability of the medium for MBH reaction

The recyclability of the reaction medium was tested under two conditions. In the first case the time of reaction was kept as 4 h. In the second case, the second run was allowed to proceed for 6 h, third run was allowed to proceed for 16 h and in the final run the time of reaction was 24 h. After completion of each step, the solvent was separated and fresh reactants were added without adding fresh DABCO to the medium. The products were isolated and purified by flash column chromatography and yield of the reaction was recorded.

### Microwave assisted organic synthesis of MBH reaction for the synthesis of monomer

Ethyl acrylate (3 mmol), aldehyde (2 mmol) and DABCO (20 mmol %) were dissolved in PEG-200. The mixture was placed in a 10 mL seamless pressure vial. The vial was closed under an argon atmosphere and placed in the cavity of a CEM microwave reactor. The reaction vial kept under 175 psi pressure, stirred at 60°C (temperature monitored by a built-in infrared sensor) and then the medium was exposed to microwave (300 W) radiation for 90s. The reaction mixture was cooled to room temperature; pH was made acidic with hydrochloric acid and the product isolated by extraction with dry ether (5×10 mL). The organic layer was combined and washed with brine (5 × 10 mL) and dried over anhydrous MgSO_4_. The solvent was evaporated and crude product was analysed by TLC and further purified by flash chromatography. The monomer was characterized by FT-IR, ^1^HNMR, ^13^CNMR, and mass spectrometry.

#### 3-ethoxy-2-methylenebut-3-en-1-ol

Colourless oil; IR (neat): *υ*_max_3405, 2878, 1725, 1634, 1439, 1207, 1069, 966, 854, 812 cm^-1^: ^1^H NMR (300 MHz, CDCl_3_, 25°C), δ (ppm): 1.26 (t, 3H, CH_3_), 3.85 (q, 2H, CH_2_CH_3_), 5.8- 6.31 (2 m, 2H, =CH_2_), 4.9 (s, 2H, CH_2_OH), 2.2 (s, 1H, OH). ^13^ C NMR (100 MHz, CDCl_3_, 25°C), δ (ppm): 166.9 (C), 140.1 (C), 124.5 (CH_2_), 61.7 (CH_2_), 61.5 (CH_2_), 14.2 (CH_3_). MS: m/z 131.08 (M^+^+1).

#### 3-ethoxy-2-methylene-1-phenylbut-3-en-1-ol

Colourless oil; IR (neat): *υ*_max_3388, 2875, 1064, 936, 884, 828 cm^-1^. ^1^H NMR (300 MHz, CDCl_3_, 25°C), δ (ppm): 1.28 (t, 3H, CH3), 1.8 (s, 1H, OH), 4.18 (q, 2H, CH_2_CH_3_), 5.17 (s, 1H, CH C_6_H_5_), 5.65-6.51 (2 m, 2H, =CH_2_), 7.35 (s, 6H, C_6_H_5_). ^13^ C NMR (100 MHz, CDCl_3_, 25°C), δ (ppm): 169.4 (C), 143.2 (C), 138.9 (C), 127.5 (2CH), 128.3 (2CH), 127.5 (CH), 70.1 (C), 62.1 (CH_2_), 14.1 (CH_3_). MS: m/z 207.10 (M^+^+1).

#### 3 Ethyl2-(hydroxyl(2-methoxypheny)methyl)acrylate

Colourless oil; IR (neat): *υ*_max_3423, 2875, 1661, 1288, 1063, 935, 885 cm^-1^. ^1^H NMR (300 MHz, CDCl_3_, 25°C), δ (ppm): 1.29 (t, 3H, CH_3_), 2.1 (s, 1H, OH), 3.81 (s, 3H, OCH_3_), 4.25 (q, 2H, CH_2_CH_3_), 5.21 (s, 1H, CH C_6_H_5_), 5.65–6.51 (2 m, 2H, =CH_2_), 6.93 (s, 6H, C_6_H_5_), 7.35 (s, 1H, CH). ^13^ C NMR (100 MHz, CDCl_3_, 25°C), δ (ppm): 169.2 (C), 156.2 (C), 137.2 (C), 128.8 (CH), 128.3 (2CH), 121.1 (CH), 112.5 (CH), 64.1 (CH), 56.2 (CH_3_), 14.1 (CH_3_). MS: m/z 237.09 (M^+^+1).

#### Ethyl2-((4-chlorophenyl)(hydroxyl)methyl)acrylate

Pale yellow oil; IR (neat): *υ*_max_3401, 2870, 1057, 935, 885, 827 cm^−1^. ^1^H NMR (300 MHz, CDCl_3_, 25°C), δ (ppm): 1.29 (t, 3H, CH_3_), 2.0 (S, 1H, OH), 4.20 (q, 2H, CH_2_CH_3_), 5.18 (S, 1H, CH), 5.72–6.27 (2 m, 2H, =CH_2_), 7.42 (s, 2H, 2CH), 7.30 (s, 2H, 2CH). ^13^ C NMR (100 MHz, CDCl_3_, 25°C), δ (ppm): 169.2 (C), 139.2 (C), 137.1 (C), 132.1 (C), 128.1 (2CH), 126.1 (2CH), 69.7 (CH_2_), 60.6 (CH_2_), 14.1 (CH_3_). MS: m/z 242.06 (M^+^+2).

#### Ethyl2-(hydroxyl (4-nitrophenyl) methyl)acrylate

Pale yellow oil; IR (neat): *υ*_max_3403, 2878, 1349, 1058, 936, 885, 816 cm^−1^. ^1^H NMR (300 MHz, CDCl_3_, 25°C), δ (ppm): 1.28 (t, 3H, CH_3_), 2.1 (s, 1H, OH), 4.19 (q, 2H, CH_2_CH_3_), 5.21 (s, 1H, CH), 5.72–6.25 (2 m, 2H, =CH_2_), 7.52 (s, 2H, 2CH), 8.21 (s, 2H, 2CH). ^13^ C NMR (100 MHz, CDCl_3_, 25°C), δ (ppm): 169.5 (C), 151.2 (C), 147.7 (C), 138.4 (C), 128.1 (2CH), 127.6 (CH_2_), 125.2 (2CH), 69.1 (CH), 61.2 (CH), 14.1 (CH_3_). MS: m/z 253.01 (M^+^+1).

### Recyclability of PEG medium for MBH reaction in presence of microwave radiation

MAOS of MBH reaction was performed as mentioned above. The product was isolated by extracting with dry ether (5 × 10 mL). After removing the respective product fresh reactants were introduced to the reaction vessel for the second run. After 90 s exposure the product was isolated using dry ether. After removing the product the PEG-200-DABCO medium was used for the third and the fourth run. After each run the product was isolated by distilling off the solvent ether. The product was further purified using flash chromatographic and HPLC techniques. After each purification step the monomer was characterized by FT-IR, ^1^HNMR, ^13^CNMR, and mass spectrometry.

## Conclusion

The present procedure to perform MBH reaction between the aldehydes and ethyl acrylate in PEG-200-DABCO medium provide an environment friendly, very simple and efficient methodology to introduce a new carbon-carbon bond in a molecule. Ability of PEG to act as an effective solvent medium is depends upon its molecular weight and as it decreases the product yield showed a significant increase. Exposure of the reaction medium to microwave radiation had a positive influence on the addition reaction in terms of overall reaction time and product yield. Reusability of the solvent-catalyst combination, higher product yield, and no use of hazardous organic solvent makes this microwave assisted synthesis in PEG-200-DABCO medium as an efficient alternative method to introduce a carbon-carbon or a carbon-heteroatom bond in a molecule.

## Conflict of interest

The authors declare that they have no conflict of interest.

## Authors' contributions

KSK & AA conceived and designed the experiments. AA performed experimental works and analyzed the data under the guidance of KSK. KSK and SG contributed reagents. All the authors have read and approved the final manuscript.

## Supplementary Material

Additional file 1 **Table S1.** Conventional DABCO catalysed Baylis-Hillman reaction in Methanol and THF. Click here for file

Additional file 2 **Table S2.** DABCO catalysed MBH reaction between formaldehyde and ethylacrylate in PEGs. Click here for file

Additional file 3 **Table S3.** DABCO catalysed MBH reaction between benzaldehyde and ethylacrylate in PEGs. Click here for file

Additional file 4 **Table S4.** DABCO catalysed MBH reaction between 2-methoxybenzaldehyde and ethylacrylate in PEGs. Click here for file

Additional file 5 **Table S5.** DABCO catalysed MBH reaction between 4-chlorobenzaldehyde and ethylacrylate in PEGs. Click here for file

Additional file 6 **Table S6.** DABCO catalysed MBH reaction between 4-nitrobenzaldehyde and ethylacrylate in PEGs. Click here for file

Additional file 7 **Table S7.** DABCO catalysed MBH reaction in PEG-200 for four subsequent runs at constant reaction time. Click here for file

Additional file 8 **Table S8.** DABCO catalysed MBH reaction in PEG-200 for four subsequent runs at extended periods of time. Click here for file

Additional file 9 **Table S9.** MAOS of MBH reaction between aldehydes and ethyl acrylate in PEG-200. Click here for file

Additional file 10 **Table S10.** MAOS: Recyclability of the medium by keeping reaction time as constant (90S) in PEG-200. Click here for file
