# Supplementary material for: Remarkable influence of microwave heating on Morita-baylis-Hillman reaction in PEG-200
Source: Chem Cent J. 2012 Apr 11;6:30. doi: 10.1186/1752-153X-6-30 (PMC3483690; doi:10.1186/1752-153X-6-30)
Supplement: Additional file 1 — Table S1. Conventional DABCO catalysed Baylis-Hillman reaction in Methanol and THF. [file 1752-153X-6-30-S1.doc]

Table 1: Conventional DABCO catalysed Baylis-Hillman reaction in Methanol and THF

| Entry | Aldehyde | Activated olefin | Methanol | | THF | |
| --- | --- | --- | --- | --- | --- | --- |
|  |  |  | Time (h) | Yield (%)^a^ | Time (h) | Yield (%)^a^ |
| 1 | HCHO | Ethylacrylate | 48 | 52 | 48 | 48 |
| 2 | C_6_H_5_CHO | Ethylacrylate | 54 | 52 | 54 | 46 |
| 3 | 2-CH_3_C_6_H_4_CHO | Ethylacrylate | 54 | 48 | 54 | 46 |
| 4 | 4-CClC_6_H_4_CHO | Ethylacrylate | 48 | 61 | 48 | 42 |
| 5 | 4-NO_2_C_6_H_4_CHO | Ethylacrylate | 38 | 50 | 38 | 44 |

^a^Isolated yields. Products were characterized by FT-IR, ^1^HNMR, ^13^C NMR and Mass spectroscopy
