# Supplementary material for: Remarkable influence of microwave heating on Morita-baylis-Hillman reaction in PEG-200
Source: Chem Cent J. 2012 Apr 11;6:30. doi: 10.1186/1752-153X-6-30 (PMC3483690; doi:10.1186/1752-153X-6-30)
Supplement: Additional file 4 — Table S4. DABCO catalysed MBH reaction between 2-methoxybenzaldehyde and ethylacrylate in PEGs. [file 1752-153X-6-30-S4.doc]

**Table 4: DABCO catalysed MBH reaction between 2-methoxybenzaldehyde and ethylacrylate in PEGs**

| **Entry** | **Aldehyde** | **Activated Olefin** | **Solvent** | **Time (h)** | **Yield (%)** |
| --- | --- | --- | --- | --- | --- |
| 1 |  |  | PEG 200 | 4 | 73 |
| 2 |  |  | PEG 300 | 4 | 52 |
| 3 |  |  | PEG 400 | 4 | 52 |
| 4 |  |  | PEG 600 | 4 | 47 |
| 5 |  |  | PEG 1000 | 4 | 46 |
