# Supplementary material for: Remarkable influence of microwave heating on Morita-baylis-Hillman reaction in PEG-200
Source: Chem Cent J. 2012 Apr 11;6:30. doi: 10.1186/1752-153X-6-30 (PMC3483690; doi:10.1186/1752-153X-6-30)
Supplement: Additional file 7 — Table S7. DABCO catalysed MBH reaction in PEG-200 for four subsequent runs at constant reaction time. [file 1752-153X-6-30-S7.doc]

**Table 7: DABCO catalysed MBH reaction in PEG-200 for four subsequent runs at constant reaction time**

| **Aldehyde** | **Run** | **1**  **Time (h)/ Isolated yield (%)** | **2** | **3** | **4** |
| --- | --- | --- | --- | --- | --- |
| Formaldehyde |  | 2/ 84 | 2/48 | 2/ 40 | 2/ 20 |
| Benzaldehyde | do | 4/ 94 | 4/50 | 4/ 46 | 4/ 32 |
| 2-methoxybenzaldehyde | do | 3/ 63 | 3/40 | 3/ 38 | 3/ 26 |
| 4-chlorobenzaldehyde | do | 4/ 92 | 4/46 | 4/ 36 | 4/ 28 |
| 4-nitrobenzaldehyde | do | 2/ 96 | 2/50 | 2/ 42 | 2/ 35 |
