# Supplementary material for: Remarkable influence of microwave heating on Morita-baylis-Hillman reaction in PEG-200
Source: Chem Cent J. 2012 Apr 11;6:30. doi: 10.1186/1752-153X-6-30 (PMC3483690; doi:10.1186/1752-153X-6-30)
Supplement: Additional file 8 — Table S8. DABCO catalysed MBH reaction in PEG-200 for four subsequent runs at extended periods of time. [file 1752-153X-6-30-S8.doc]

**Table 8: DABCO catalysed MBH reaction in PEG-200 for four subsequent runs at extended periods of time**

| **Aldehyde** | **Run** | **1**  **Time (h)/ Isolated yield (%)** | **2** | **3** | **4** |
| --- | --- | --- | --- | --- | --- |
| Formaldehyde |  | 2/ 84 | 6/58 | 16/ 52 | 24/ 50 |
| Benzaldehyde | do | 4/ 94 | 6/68 | 16/ 62 | 24/ 68 |
| 2-methoxybenzaldehyde | do | 3/ 63 | 6/58 | 16/ 46 | 24/ 42 |
| 4-chlorobenzaldehyde | do | 4/ 92 | 6/75 | 16/ 70 | 24/ 65 |
| 4-nitrobenzaldehyde | do | 2/ 96 | 6/74 | 16/ 72 | 24/ 70 |
