# Supplementary material for: Remarkable influence of microwave heating on Morita-baylis-Hillman reaction in PEG-200
Source: Chem Cent J. 2012 Apr 11;6:30. doi: 10.1186/1752-153X-6-30 (PMC3483690; doi:10.1186/1752-153X-6-30)
Supplement: Additional file 9 — Table S9. MAOS of MBH reaction between aldehydes and ethyl acrylate in PEG-200. [file 1752-153X-6-30-S9.doc]

**Table 9: MAOS of MBH reaction between aldehydes and ethyl acrylate in PEG-200**

| **Entry** | **Aldehyde** | **Activated Olefin** | **Solvent medium** | **Time (S)** | **Yield (%)^b^** |
| --- | --- | --- | --- | --- | --- |
| 1 | Formaldehyde |  | PEG-200 | 90 | 92 |
| 2 | Benzaldehyde |  | PEG-200 | 90 | 88 |
| 3 | 2-methoxybenzaldehyde |  | PEG-200 | 90 | 90 |
| 4 | 4-chlorobenzaldehyde |  | PEG-200 | 90 | 90 |
| 5 | 4-nitrobenzaldehyde |  | PEG-200 | 90 | 89 |

^b^Isolated yields. Products were characterized by FTIR, ^1^H, ^13^C NMR and Mass spectroscopy
