# Supplementary material for: Remarkable influence of microwave heating on Morita-baylis-Hillman reaction in PEG-200
Source: Chem Cent J. 2012 Apr 11;6:30. doi: 10.1186/1752-153X-6-30 (PMC3483690; doi:10.1186/1752-153X-6-30)
Supplement: Additional file 10 — Table S10. MAOS: Recyclability of the medium by keeping reaction time as constant (90S) in PEG-200. [file 1752-153X-6-30-S10.doc]

**Table 10: MAOS: Recyclability of the medium by keeping reaction time as constant (90S) in PEG-200**

| **Aldehyde** | **Run/ Reaction time (90s)** | **1**  **Isolated yield (%)** | **2** | **3** | **4** |
| --- | --- | --- | --- | --- | --- |
| Formaldehyde | do | 92 | 76 | 78 | 72 |
| Benzaldehyde | do | 88 | 74 | 74 | 66 |
| 2-methoxybenzaldehyde | do | 90 | 85 | 73 | 68 |
| 4-chlorobenzaldehyde | do | 90 | 80 | 68 | 62 |
| 4-nitrobenzaldehyde | do | 89 | 78 | 68 | 60 |
